# Exploring the relative contributions of learning motivations and test perceptions to autonomous English as a foreign language learning and achievement

**DOI:** 10.3389/fpsyg.2023.1059375

**Published:** 2023-01-25

**Authors:** Xiaohua Liu, Manxia Dong

**Affiliations:** ^1^The Chinese University of Hong Kong, Shenzhen, China; ^2^Sichuan International Studies University, Chongqing, China

**Keywords:** language learning motivation, test perception, learning practice, learning outcome, English as a foreign language, secondary education, regression analysis

## Abstract

Past studies on the contributions of language learning motivations and test perceptions to language learning have been conducted relatively independently, with few simultaneously gauging the relative effects of these two types of variables on learning behaviors and outcome. In contexts where testing plays a significant role in language education, it is argued that both types of variables are likely to influence language learning. Through a series of multiple regression analyses, this study juxtaposed the relative effects of three types of language learning motivation (i.e., integrative, development and requirement motivation) and two types of perception of a high-stakes English test on Chinese high school students’ (*n* = 3,105) EFL learning practice and achievement, casting fresh lights on the motivational factors that may drive EFL learning. More specifically, it was found that integrative and development motivations were the major drives behind students’ overall effort expenditure on EFL learning for Year 1 students. For students from higher grades who were more closely confronted with the test, however, the effect of development motivation diminished and that of perceived test validity increased. The same pattern applied to students’ reported learning achievement. The motivational profiles behind each specific type of learning practice and their variational patterns across grades were also found to differ. Implications for both research and educational practice are discussed.

## 1. Introduction

Due to its significant impact on the process and product of learning, learning motivation has been a lasting research focus in fields of both language learning and general education. Researchers also distinguished between different types of language learning motivation, including motivation associated with the target language (e.g., integrative vs. instrumental orientation, and interest in foreign languages), with the learning situation (e.g., teachers and courses) and with learners themselves (e.g., self-efficacy, language use anxiety, and desire for achievement; Dörnyei, 2005; Gardner, 2010). Test-oriented learning motivation can be said to fit into the category related to learning situation, as tests are often considered to be part of or closely association with a curriculum. In many education systems, such as China, testing has been taking such a prominent role that its influences on teaching and learning (now commonly known as washback) were found to be widespread and pervasive (Shohamy, 2001; Cheng and Qi, 2006), prompting a surge of washback studies, including a few recent ones about students’ perceptions of language tests and their effects on language learning practice (Xie and Andrews, 2013; Zhan and Andrews, 2014; Liu and Yu, 2021).

To date, however, studies probing into the roles language learning motivation (LLM) and test perception (TP) play in language learning have been conducted relatively independently. It is contended that in contexts where language education is infiltrated with a testing culture, simultaneously investigating both types of variables in relation to learning in one study is of both theoretical and pedagogical interests. This is because the results would show a more complete picture about the motivational profile behind students’ learning practice, and thus would contribute to future model building. The results can also benefit language education, as teachers, curriculum and test designers as well as policy makers can more accurately pinpoint and weigh the major types of motivational factors driving students’ specific learning practice, and take appropriate measures to facilitate desirable practice and to reduce undesirable one.

### 1.1. Chinese high school EFL education and the role of *Gaokao*

China’s secondary education consists of a 3-year junior middle schooling and another 3 years of senior middle schooling. At the end of each stage, students are required to sit a suite of tests, and the results will largely determine the type of education at the next level they are qualified for. The test battery at the end of senior middle schools is called National Matriculation Test, also widely known as *Gaokao* in Chinese. It comprises three compulsory tests (i.e., Chinese, English and mathematics, which have the largest weight in the total score) and several operational tests on other subjects (e.g., physics, chemistry, geography, etc.,). In recent years, as many as around 10 million high school graduates would sit *Gaokao* annually. Although about 75% of them would successfully enter higher education, the total score they achieve in *Gaokao* would determine the type of universities or colleges they can attend. Consequently, this test is seen by millions of test takers and their families as the battleground where they have to compete for better education opportunities.

The exceptionally high stakes of the test permeate many aspects of the society (Cheng and Qi, 2006). In classrooms, teachers and students have also been reported to teach and learn to the test, particularly in the last year (i.e., Senior III), to boost test scores (Qi, 2005, 2007). As one of the three compulsory tests, the English test of *Gaokao* plays a significant role in the competitive selection process of university admissions. For most regions of the country, it mainly consists of a paper-and-pencil test, which has a full mark of 150 and is routinely composed of four parts: listening, reading, writing and language knowledge (i.e., grammar, vocabulary, and pragmatic knowledge). Except for writing, the other three parts mostly adopt objective tasks, such as multiple-choice questions and banked cloze. Throughout the 3-year schooling, achievement tests (such as mid-term and end-of-term exams) are usually modeled on this format. During Senior III, test drilling normally intensifies, and many schools would organize monthly mock tests which serve as both dry runs and monitors of test preparation progress.

As an academic stage that is supposed to develop students’ knowledge and abilities but at the same time unavoidably heavily influenced by *Gaokao*, high schooling in China is a perfect setting to investigate simultaneously the influences LLM and TP on language learning. As teaching and learning to the test normally intensify with the approach of test date, it also provides the opportunity to examine how different temporal distances from the test may mediate the effects of LLMs and TPs.

### 1.2. Language learning motivations

Rooted in social psychology, the socio-educational model of LLM proposed by Gardner and Lambert (1972) has exerted far-reaching impact in the field of LLM, particularly their classic distinction between integrative and instrumental motivation (see also Gardner, 2010). According to them, integrative motivation is concerned with an intrinsic interest in the target language, the community and culture of its speakers, as well as a willingness to acquire the language for the purpose of interacting and identifying with members of that community; instrumental motivation, by contrast, concerns the inclination of utilizing the target language as a means to achieve pragmatic goals, such as monetary rewards, promotion and social recognition (Gardner and Lambert, 1972). This differentiation is similar to the ideal versus ought-to L2 self dichotomy proposed by Dörnyei (2005) and the differentiation between intrinsic and extrinsic motivations made by achievement goal theories (e.g., Self-Determination Theory) from educational psychology (Ryan and Deci, 2017).

According to Dörnyei (2005), ideal L2 selves represent language learners’ visions of themselves in the future in relation to the target language (e.g., speaking the language with people from the L2 community) and thus belongs to the integrative type of LLM; ought-to L2 selves entail responsibilities imposed by external entities or systems that learners think they ought to possess, and therefore are instrumental in general (Dörnyei, 2005). From the perspective of learning behavior, achievement goal theories posit that students who are intrinsically motivated tend to focus on learning new knowledge and skills (Ames, 1992) and are more likely to take on challenging tasks and engage in deep learning strategies (Fenollar et al., 2007; Hulleman et al., 2008; Senko et al., 2013). In contrast, extrinsically motivated students are more likely to pursue external standards and rewards (Ames, 1992; Hulleman et al., 2008), and have the tendency to employ surface learning strategies and engage in intensive test practice (Senko et al., 2013; Liu and Yu, 2021).

Scholars also noted that, however, depending on the extent to which external standards or norms are integrated into personal values and goals, instrumental or extrinsic motivations can vary in terms of internalization and regulation (Ryan and Deci, 2017). Therefore, students’ who have fully aligned instrumental goals such as studying for a better career with their personal value would be more committed to learning than those driven by completely external goals such as gaining better test scores to please parents or teachers; instrumental goals of the former group, according to Ryan and Deci (2017), actually come closer to intrinsic motivations. In a similar vein, Dörnyei (2005) argues that instrumentality concerning career development or professional success is more related to an ideal L2 self. It is different from non-internalized instrumental motivation imposed by external requirements, which is associated with an ought-to L2 self. According to Dörnyei (2005), the former is more likely than the latter to motivate learners to make sustained commitment to learning the target language.

Generally in consistence with these assumptions, integrative motivation and ideal L2 self, as compared with instrumental motivation and ought-to L2 self, have been frequently found to bear stronger positive relations with effort expenditure in language learning, including overall use of metacognitive strategies (Wu, 2007; Baleghizadeh and Rahimi, 2011), intended learning effort (Csizér and Kormos, 2009; Papi, 2010), learning persistence (Gardner et al., 2004) and intention to continue to learn (Hernández, 2006; McEown et al., 2014). McEown et al. (2014) also found that students’ identified regulation, one type of internalized instrumentality, had higher contribution than their intrinsic motivation did to their intention to continue to learn, whereas other less internalized instrumental motives had little contribution to this intention.

It shall be mentioned that these studies of the effects of different LLMs on language learning mostly focused on overall learning effort and strategy engagement, without looking at specific learning behaviors. In contrast, achievement goal studies in educational psychology have extensively investigated the effects of different goal orientations on different learning behaviors, such as deep versus surface learning strategies. These studies generally found that while intrinsic goals tend to be positive predictors of deep or meaningful learning strategies (*β* = 0.20–0.53), extrinsic goals are more often positive determinants of surface learning strategies (*β* = 0.14–0.41; Greene and Miller, 1996; Fenollar et al., 2007; Senko et al., 2013). Meanwhile, Senko et al. (2013) found that extrinsic goals positively contributed to students’ test-oriented activities (*β* = 0.22–0.27), while intrinsic goals prompted students to conduct more interest-based activities (*β* = 0.16).

In a language learning setting, Liu and Yu (2021) examined the relationships between two goal orientations (intrinsic vs. extrinsic or test) and three types of learning practice (i.e., normal language learning, rote learning, and test drilling). It was found that intrinsic goals significantly contributed to students’ normal language learning (*β* = 0.44) and rote learning (*β* = 0.14), while extrinsic or test-oriented goals significantly contributed to their test drilling (*β* = 0.44) and rote learning (*β* = 0.46). In this study, Liu and Yu (2021) note that while rote learning represents a typical surface learning strategy, test drilling may actually involve deep learning strategies, as drilling for a language test at the tertiary level extensively require sophisticated language processing and reasoning skills. However, test drilling is also generally regarded as useless language learning practice, since past studies revealed that although it may be effective in familiarizing students with test format and test-taking strategies—and thus boost test scores to some extent—it is not effective in improving real language abilities (Qi, 2007; Green, 2007b; Winke and Lim, 2017). In many situations, it is also dismissed as negative washback effect that inhibits normal language development (Qi, 2005, 2007; Cheng and Qi, 2006). From an educational standpoint, therefore, it is necessary to differentiate such undesirable learning practice from other more desirable ones, and to pinpoint the motivational forces behind each one of them.

In terms of the relationships between different LLMs and learning outcome, Hernández (2006) found that university students’ integrative motivation in learning Spanish as a foreign language in the United States significantly predicted their scores on a speaking test (*β* = 0.38), while both internalized instrumental motivation (i.e., learning the language for personal development) and non-internalized one (i.e., learning the language to fulfill university language requirement) had little contribution to their test scores. Shaikholeslami and Khayyer (2006) regressed Iranian university students’ GPA in English exams onto their amotivation, three types of extrinsic motivation and three types of intrinsic motivation in English learning. They found that only intrinsic motivation concerning personal stimulation (*β* = 0.19) positively predicted GPA with statistical significance, while amotivation (*β* = −0.22), introjected extrinsic motivation for ego enhancement (*β* = −0.22) and intrinsic motivation for knowledge acquisition (*β* = −0.25) negatively predicted GPA with statistical significance; identified extrinsic motivation for personal development showed sizeable but statistically nonsignificant contribution (*β* = 0.17, *p* = 0.05), and the contributions from both extrinsic motivation for external regulation and intrinsic motivation for accomplishment were small and statistically nonsignificant.

Other studies also examined the relations between intrinsic and extrinsic motivations and students’ self-evaluation of their language proficiency, and found that intrinsic motivation showed stronger positive relations with self-evaluated proficiency than extrinsic motivation (including its subtypes) did (MacIntyre and Blackie, 2012; McEown et al., 2014). In short, past studies generally discovered that intrinsic and integrative motivations appeared to be fairly consistent positive contributors of language learning achievement, whereas non-internalized instrumental motivations had little or even negative contributions. Internalized instrumental motivations such as for personal development may also positively contribute to learning achievement, but its contribution may not be as strong as that of intrinsic or integrative motivation.

### 1.3. Test perceptions

Apart from LLMs, test perceptions may exert their own influences on learning practice, especially in a washback context where an influential test has a profound impact on aspects of teaching and learning. Green (2007a) highlighted two contributing factors of test-oriented practice: perceptions of test importance and difficulty. According to him, only tests that are considered both important and challenging (but achievable) are likely to drive students to study for them. In context of a high-stakes test, however, it has also been found that students’ strong desire to succeed in the test would prompt them to engage in intense test drilling, regardless of their evaluation of test difficulty. For example, Liu and Yu (2021) found that students’ test-directed motivation correlated more highly with test drilling (*r* = 0.46) than did their evaluation of self-efficacy in those tasks (*r* = 0.31); further structural equation modeling revealed that test-oriented motivation directly predicted test practice with statistical significance (*β* = 0.48), and while self-efficacy also had a direct effect on test practice (*β* = 0.26), it did not play any role in mediating test-oriented motivation’s prediction of test drilling.

Xie and Andrews (2013) modeled the relationships between students’ perception of a high-stakes English language test’s skill demands, instrumental motives for taking the test, perceived value of the test, expectation for success, and their use of test preparation strategies. It was found that the more students acknowledged that the test assessed the listed language skills (which were taken from the testing syllabus), the more they would employ test preparation strategies (*β* = 0.39), which were different from those language skills. The standardized effect was much larger than that of either instrumental test motivations (*β* = 0.003) or perceived test value (*β* = 0.14) on use of test preparation strategies. Further analysis also found that students’ recognition of the language skills being tested had little effect on their engagement in real language development activities (*β* = 0.08, *p* > 0.05; Xie, 2015). These findings led the authors to conclude that students’ identification of the construct of a test does not necessarily lead them to engage in desirable learning activities developing those skills, but may stir up their passion for test drilling instead (Xie and Andrews, 2013; Xie, 2015).

Few studies examined the direct contributions of test perceptions to learning achievement. However, a number of studies investigated the effect of test preparation or drilling on score gain. While some of them found that intense test drilling did help boost test scores (Farnsworth, 2013; Xie, 2013), others revealed little of such effect on score gain (Green, 2007b; Winke and Lim, 2017). Meanwhile, authors of these studies generally suspected that focused test drilling mainly helped students with a familiarity of test format and test-taking strategies rather than real language development, as mentioned earlier.

To encapsulate, past studies generally found that internalized motivations such as integrative motivation and motivation for personal development tend to be more adapted than non-internalized motivations (such as instrumentality for external requirements) in facilitating language learning and learning achievement. However, these studies were mostly conducted without considering the influences of testing and test-related perceptions or attitudes. On the other hand, studies which focused on test perceptions found that perceived test validity and importance would both drive students to engage in test-oriented activities, which themselves may only enhance testwiseness instead of real language development. These washback studies also failed to consider the effects of different LLMs in addition to those of test perceptions. Moreover, few of these studies distinguished between different types of learning practice, which may be differentially motivated by different LLMs and TPs. In short, we have little knowledge about the relative contributions of LLMs and TPs to different language learning behaviors and achievement.

Another notable point is that those studies on the relationship between LLMs or TPs and learning behaviors and outcome seldom took into account the role of temporality. MacIntyre et al. (2009) note that the quality of LLMs such as possible selves are likely to change with the approach of important events or dates. Based on interview and diary data, Zhan (2009) found that students experienced changes in their visions of possible selves before and after a high-stakes English language test, and such changes in selves also triggered changes in their English language learning behaviors outside class. Therefore, we may speculate that the predictability of LLMs and TPs for language learning practice could be different at different time points in relation to an important test.

Due to this and the points discussed earlier, this study endeavored to gauge the relative effects of different LLMs and TPs on different types of language learning practice and achievement, and how these effects may vary across students who are differentially distanced from a high-stakes language test. Therefore, three sets of research questions were asked of the study:

RQ1: For each grade, which LLM and TP variables constitute the strongest predictors of students’ overall investment in English language learning outside class (as measured by learning time and average frequency of English language learning practice)? Do their contributions to this overall investment vary from lower to higher grades? If yes, how?

RQ2: For each grade, which LLM and TP variables constitute the strongest predictors of students’ engagement in each type of learning practice? Do their contributions to each type of learning practice vary from lower to higher grades? If yes, how?

RQ3: For each grade, which LLM and TP variables constitute the strongest predictors of students’ reported achievement in English language learning? Do their contributions to reported achievement vary from lower to higher grades? If yes, how?

## 2. Methods

### 2.1. Participants

In this study, a questionnaire survey was conducted with 3,278 senior middle school students from six schools, which were all located in a southwest city of China. The six schools differed in their level of jurisdiction and social status (see Table 1). Altogether 3,215 copies of the questionnaire were returned from the students. After a careful screening, 110 copies were regarded as being invalid and excluded from subsequent data analyses due to over 10% of unanswered questions each or obvious response patterns. For the remaining questionnaires, background information of their respondents (as grouped by grade) are shown in Table 1. We can see that the three groups’ profiles were rather similar, except for their mean age, which increased with the ascending of grade level.

**Table 1:** Questionnaire respondents’ background information.

		Senior I (*n* = 1,199)	Senior II (*n* = 1,098)	Senior III (*n* = 808)
Gender	Female	671 (56.0%)	566 (51.5%)	466 (57.7%)
	Male	528 (44.0%)	532 (48.5%)	342 (42.3%)
Age	≤15	177 (14.8%)	6 (0.5%)	1 (0.1%)
	16	740 (61.7%)	130 (11.8%)	2 (0.2%)
	17	267 (22.3%)	670 (61.0%)	106 (13.1%)
	18	14 (1.2%)	274 (25.0%)	505 (62.5%)
	≥19	1 (0.1%)	18 (1.6%)	194 (24.0%)
Type of senior middle school attending	Town ordinary	154 (12.8%)	158 (14.4%)	152 (18.8%)
	County ordinary	93(7.8%)	123 (11.2%)	87 (10.8%)
	District ordinary	162 (13.5%)	214 (19.5%)	175 (21.7%)
	District key	241 (20.1%)	250 (22.8%)	151 (18.7%)
	City key	376 (31.4%)	189 (17.2%)	151 (18.7%)
	City top	173 (14.4%)	164 (14.9%)	92 (11.4%)
Time started learning English	Kindergarten	41 (3.4%)	58 (5.3%)	27 (3.3%)
	Primary school	526 (43.9%)	457 (41.6%)	363 (44.9%)
	Junior middle school	632 (52.7%)	583 (53.1%)	418 (51.7%)
Type of junior middle school attended	Town	464 (38.7%)	404 (36.8%)	305 (37.7%)
	District ordinary	259 (21.6%)	234 (21.3%)	178 (22.0%)
	District key	274 (22.9%)	268 (24.4%)	207 (25.6%)
	Provincial/city key	191 (15.9%)	182 (16.6%)	116 (14.4%)
	Other	11 (0.9%)	10 (0.9%)	2 (0.2%)

### 2.2. Instrument

The student questionnaire was written in Chinese and had multiple sections (with a total of 87 items), which focused on aspects of students’ English language learning. The parts reported in this article include a language learning motivation (LLM) scale, a test perception (TP) scale and an out-of-class language learning practice (LLP) scale.

The LLM scale consisted of three subscales (with 11 items in total): Integrative Motivation (LLM-Integrative, *α* = 0.81–0.83 across grades), Development Motivation (LLM-Development, *α* = 0.82–0.85) and Requirement Motivation (LLM-Requirement, *α* = 0.66–0.71). These items were adapted from the *Attitude/Motivation Test Battery* (Gardner et al., 1997) and the EFL learning motivation questionnaire developed specifically with Chinese students by Gao et al. (2007). Each item within the scale described one purpose for EFL learning. Respondents were supposed to rate their agreement with each purpose on a five-point scale (i.e., “agree,” “somewhat agree,” “not sure,” “somewhat disagree” to “disagree”).

LLM-Integrative had four items, which tap into learners’ intrinsic urge to interact with people speaking the target language and interest in their cultures (e.g., “For being able to understand the cultures and traditions of major English-speaking countries” and “For being able to make friends with foreigners”). LLM-Development comprised another four items, which address students’ motives for promoting personal development through English learning (e.g., “For gaining better opportunities for education and development in future” and “For gaining an edge in job hunting in future”) and represent internalized instrumentality (Dörnyei, 2005; Ryan and Deci, 2017). LLM-Requirement had three items, which describe learning English for immediate achievements and for fulfilling obligations or requirements (e.g., “For meeting my parents’ expectations” and “For gaining a higher score in the English test of *Gaokao* because it is a compulsory test”). This subscale represents non-internalized instrumentality that is externally imposed (Dörnyei, 2005; Ryan and Deci, 2017).

The TP scale (with six items in total) included the TP-Validity (*α* = 0.79–0.82) and TP-Importance (*α* = 0.72–0.77) subscales. Each item was also rated with the same Likert-style agreement scale and was constructed based on interviews with students (Dong, 2016) as well as references to questionnaires from previous studies (Cheng, 2005; Gu, 2007). TP-Validity had three items, which probe into students’ perceptions of *Gaokao*’s validity and reliability (e.g., “*Gaokao* will be a valid examination of my English learning achievements during my senior middle school” and “*Gaokao* results accurately and objectively reflect senior middle school students’ English language proficiency”). TP-Importance also had three items, which measure how important the test is considered by students (e.g., “Gaining a high score in *Gaokao* is important for my confidence in English learning in future” and “Gaining a high score in *Gaokao* is important to me, because it helps me maintain a good image in front of my classmates, teachers and family members”).

The LLP scale (with a total of 18 items) was constituted by four subscales: Amusement Learning Activities (LLP-Amusement, *α* = 0.71–0.74), Communicative Learning Activities (LLP-Communicative, *α* = 0.84–0.88), Curriculum-based Learning Activities (LLP-Curriculum, *α* = 0.83–0.84) and Test Drilling Activities (LLP-Test, *α* = 0.83–0.85). Items from this scale were also informed by student interviews, diary entries (Dong, 2016), and previous questionnaires (Cheng, 2005; Gu, 2007). In rating each item, respondents needed to consider the frequency of the activity described by it and to choose between five options: “always,” “often,” “sometimes,” “seldom” and “never.” LLP-Amusement had two items, describing activities conducted for enjoyment (i.e., “Listening to songs in English” and “Watching motives and TV shows in English”). LLP-Communicative contained seven learning activities, which all had strong communicative features (e.g., “Writing notes, letters or emails in English” and “Communicating with my classmates and teachers in English”). Five items constituted the LLP-Curriculum subscale, which depicted activities associated with teaching content or curriculum (e.g., “Reviewing what has been taught in class” and “Doing exercises from the textbook”). There were four items in LLP-Test, which were all about drilling for *Gaokao* (e.g., “Practicing *Gaokao* past papers” and “Reciting or memorizing *Gaokao* model writings”).

Apart from the three major scales, there was one question asking about the daily average time spent on English learning outside class. Respondents were supposed to choose from five options: “0 h,” “about 0.5 h,” “about 1 h,” “about 1.5 h,” and “about 2 h or more.” Finally, the questionnaire asked participants to report the range of scores they achieved in school-based English language exams around the time of the survey. Five score ranges were provided: “≤75,” “76 ~ 90,” “91 ~ 105,” “106 ~ 119,” and “≥120.” These ranges were set based on discussions with English teachers from the six schools, who generally agreed that they corresponded to performance levels ranging from “very poor,” “poor,” “average,” “good” to “very good.”

### 2.3. Data collection and analysis

The questionnaire survey was conducted in May and June of 2014, which were near the end of the academic year. All questionnaires were completed in class. More specifically, the English teacher of each class first briefed the students about the survey, including its purpose, major content, and its voluntary and anonymous nature. Afterwards he or she handed out hard copies of the questionnaire. Students who opted not to participate were given other teaching tasks to complete. Once being finished, the questionnaires were collected by the teacher, who then mailed them back to the researchers.

Questionnaire responses were later manually entered into computer. As questions from the main sections all had five options, answers to them were coded from 1 to 5, with 1 representing the smallest or most negative option in meaning. Once finished, the digital data were then systematically checked for entry errors, missing values, outliers and item normality. Only 6 cases from the 3,105 questionnaires had a few missing values, which were replaced with item means. Items were also found to be generally normally distributed (see the Supplemental material for item-level descriptive statistics). Across the three student groups, the subscales of LLM, TP and LLP had *α* values between 0.66 and 0.88, with a mean of 0.80, indicating that these subscales had acceptable to excellent levels of reliability. Item scores within each subscale were then averaged, generating a set of new items representing the latent variables under investigation. Besides, scores of all items from the LLP scale were averaged to create a new variable representing the average frequency of English language learning practice.

The newly generated variables were checked for potential outliers, univariate and multivariate normality, collinearity and multicollinearity. Table 2 displays their descriptive information. It was found that across grades, most variables were normally distributed. However, two variables—LLM-Development and LLP-Communicative—caught our attention, as they generally had skewness and kurtosis values notably deviant from the range of ± 1, especially for the kurtosis of LLP-Communicative from Senior II. Histograms of these two variables showed that they were indeed abnormally skewed and peaked. Due to these normality issues, they were log-transformed (Tabachnick and Fidell, 2013). After preliminary analyses, nevertheless, it was found that regression results with and without transformed variables showed none to only very small differences. This concurred earlier suggestion that large sample sizes help reduce the negative impact of normality issues (Tabachnick and Fidell, 2013). Therefore, in the Results section we will report only results from analyses with untransformed variables for interpretation consistency.

**Table 2:** Descriptive statistics of independent and dependent variables.

	Senior I				Senior II				Senior III			
	*M*	SD	Skewness	Kurtosis	*M*	SD	Skewness	Kurtosis	*M*	SD	Skewness	Kurtosis
LLM-integrative	3.52	1.03	−0.61	−0.31	3.42	1.15	−0.47	−0.66	3.41	1.12	−0.51	−0.60
LLM-development	4.17	0.87	−1.19	1.13	4.07	0.97	−1.11	0.67	4.07	0.99	−1.23	1.06
LLM-requirement	3.66	1.11	−0.67	−0.40	3.75	1.10	−0.81	−0.15	3.61	1.14	−0.70	−0.36
TP-validity	3.41	1.08	−0.51	−0.46	3.18	1.17	−0.32	−0.82	3.10	1.20	−0.24	−0.97
TP-importance	3.38	1.08	−0.44	−0.52	3.25	1.17	−0.40	−0.71	3.19	1.16	−0.36	−0.73
LLP-time	2.40	0.96	−0.56	0.13	2.45	1.08	0.62	−0.10	2.45	1.10	0.72	−0.02
LLP-Av. Freq.	2.87	0.63	−0.08	0.12	2.83	0.65	−0.02	0.08	2.99	0.66	−0.14	0.23
LLP-amusement	3.36	1.01	−0.15	−0.57	3.32	1.05	−0.09	−0.65	3.36	1.05	−0.20	−0.56
LLP-communicative	1.81	0.70	1.15	1.55	1.68	0.69	1.52	2.79	1.80	0.81	1.31	1.46
LLP-curriculum	3.66	0.87	−0.73	0.26	3.45	0.91	−0.53	−0.14	3.38	0.89	−0.38	−0.11
LLP-test	2.66	0.95	0.18	−0.44	2.88	1.03	0.05	−0.57	3.42	0.99	−0.44	−0.23
Achievement	2.66	1.32	0.24	−1.08	2.44	1.27	0.37	−1.04	2.54	1.24	0.28	−0.97

To answer the three research questions, a series of standard multiple regressions were conducted with data from each student group, a type of analysis suitable for comparing the relative contributions among a set of predictors to a criterion variable (Tabachnick and Fidell, 2013). Considering the normality issues mentioned earlier, all regression analyses were accompanied by bootstrapping. Analyses were conducted through SPSS 23.0.

## 3. Results

**Figure 1:**
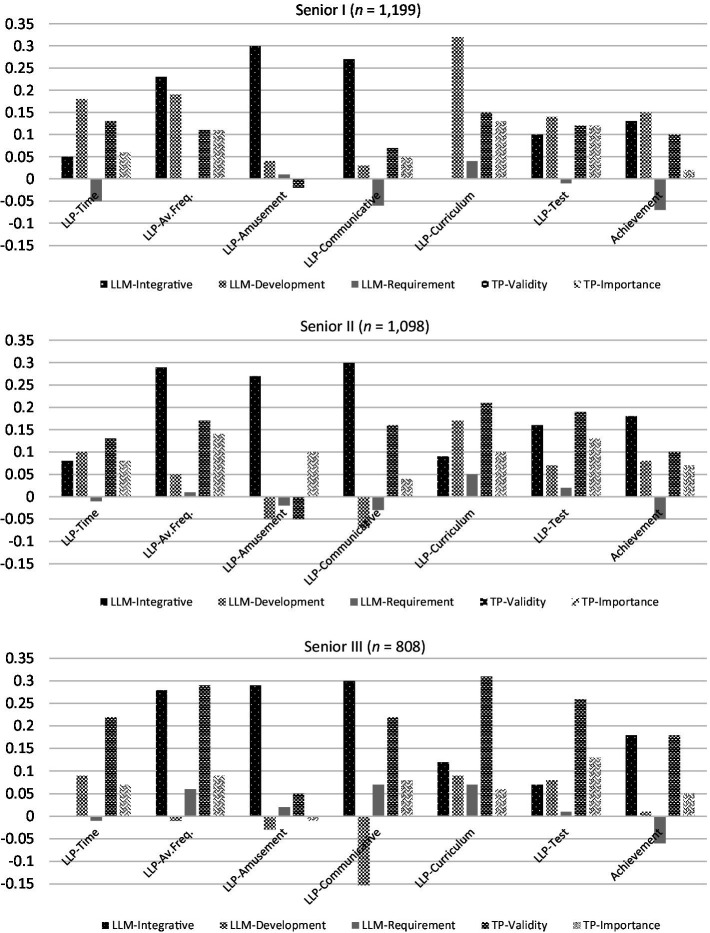
Standard regression coefficients.

Inter-correlations between the independent and dependent variables in the regression analyses ranged from marginal to medium in size (see Table 3). Table 4 presents the regression results for each grade, and Figure 1 graphically shows the variation patterns of standard regression coefficients.

**Table 3:** Pearson correlations between independent and dependent variables.

		LLP-time	LLP-Av. Freq.	LLP-amusement	LLP-communicative	LLP-curriculum	LLP-test	Achievement
Senior I	LLM-integrative	0.20^**^	0.38^**^	0.32^**^	0.31^**^	0.22^**^	0.22^**^	0.24^**^
	LLM-development	0.27^**^	0.38^**^	0.20^**^	0.21^**^	0.41^**^	0.27^**^	0.25^**^
	LLM-requirement	−0.02	0.01	−0.03	−0.07^*^	0.10^**^	0.02	−0.06^*^
	TP-validity	0.22^**^	0.28^**^	0.07^*^	0.16^**^	0.32^**^	0.25^**^	0.18^**^
	TP-importance	0.18^**^	0.26^**^	0.06	0.12^**^	0.32^**^	0.24^**^	0.12^**^
Senior II	LLM-integrative	0.20^**^	0.40^**^	0.26^**^	0.32^**^	0.27^**^	0.28^**^	0.28^**^
	LLM-development	0.23^**^	0.34^**^	0.13^**^	0.18^**^	0.35^**^	0.29^**^	0.25^**^
	LLM-requirement	0.02	0.03	−0.04	−0.03	0.09^**^	0.06	−0.04
	TP-validity	0.23^**^	0.33^**^	0.04	0.22^**^	0.35^**^	0.32^**^	0.21^**^
	TP-importance	0.21^**^	0.33^**^	0.13^**^	0.17^**^	0.32^**^	0.31^**^	0.21^**^
Senior III	LLM-integrative	0.12^**^	0.36^**^	0.29^**^	0.26^**^	0.25^**^	0.22^**^	0.25^**^
	LLM-development	0.20^**^	0.29^**^	0.15^**^	0.09^**^	0.30^**^	0.27^**^	0.20^**^
	LLM-Requirement	0.01	0.04	−0.03	0.05	0.07^*^	0.02	−0.07^*^
	TP-Validity	0.29^**^	0.40^**^	0.11^**^	0.26^**^	0.41^**^	0.38^**^	0.25^**^
	TP-Importance	0.21^**^	0.31^**^	0.08^*^	0.20^**^	0.29^**^	0.31^**^	0.18^**^

^*^p < 0.05, ^**^p < 0.01.

**Table 4:** Results of standard multiple regressions.

			LLP-time	LLP-Av. Freq.	LLP-amusement	LLP-communicative	LLP-curriculum	LLP-test	Achievement
Senior I	*β*	LLM-integrative	0.05	0.23^**^	0.30^**^	0.27^**^	0.00	0.10^**^	0.13^**^
		LLM-development	0.18^**^	0.19^**^	0.04	0.03	0.32^**^	0.14^**^	0.15^**^
		LLM-requirement	−0.05	0.00	0.01	−0.06	0.04	−0.01	−0.07
		TP-validity	0.13^**^	0.11^**^	−0.02	0.07^*^	0.15^**^	0.12^**^	0.10^**^
		TP-importance	0.06	0.11^**^	0.00	0.05	0.13^**^	0.12^**^	0.02
	*F*		26.41^***^	66.06^***^	26.80^***^	29.36^***^	69.65^***^	31.21^***^	24.40^***^
	*R* ^2^		0.10	0.22	0.10	0.11	0.23	0.12	0.09
	*R*^2^ adjusted		0.10	0.21	0.10	0.11	0.22	0.11	0.09
Senior II	*β*	LLM-integrative	0.08^*^	0.29^**^	0.27^**^	0.30^**^	0.09^**^	0.16^**^	0.18^**^
		LLM-development	0.10^**^	0.05	−0.05	−0.07	0.17^**^	0.07	0.08^*^
		LLM-requirement	−0.01	0.01	−0.02	−0.03	0.05	0.02	−0.05
		TP-validity	0.13^**^	0.17^**^	−0.05	0.16^**^	0.21^**^	0.19^**^	0.10^**^
		TP-importance	0.08^*^	0.14^**^	0.10^*^	0.04	0.10^*^	0.13^**^	0.07^*^
	*F*		20.47^***^	64.81^***^	17.90^***^	31.05^***^	52.93^***^	44.09^***^	27.01^***^
	*R* ^2^		0.09	0.23	0.08	0.12	0.20	0.17	0.11
	*R*^2^ adjusted		0.08	0.22	0.07	0.12	0.19	0.16	0.11
Senior III	*β*	LLM-integrative	0.00	0.28^**^	0.29^**^	0.30^**^	0.12^**^	0.07	0.18^**^
		LLM-development	0.09^*^	−0.01	−0.03	−0.18^**^	0.09	0.08	0.01
		LLM-requirement	−0.01	0.06	0.02	0.07^*^	0.07^*^	0.01	−0.06
		TP-validity	0.22^**^	0.29^**^	0.05	0.22^**^	0.31^**^	0.26^**^	0.18^**^
		TP-importance	0.07	0.09^*^	−0.01	0.08^*^	0.06	0.13^**^	0.05
	*F*		16.97^***^	51.53^***^	14.74^***^	25.12^***^	42.09^***^	34.82^***^	18.84^***^
	*R* ^2^		0.10	0.24	0.08	0.14	0.21	0.18	0.11
	*R*^2^ adjusted		0.09	0.24	0.08	0.13	0.20	0.17	0.10

LLM = language learning motivation, LLP = language learning practice, Av. Freq. = average frequency; *β* = standardized regression coefficient; results of significance testing of coefficients were based on 1,000 bootstrap samples; ^*^p < 0.05, ^**^p < 0.01, ^***^p < 0.001.

*RQ1*: For each grade, which LLM and TP variables constitute the strongest predictors of students’ overall investment in English language learning outside class (as measured by learning time and average frequency of English language learning practice)? Do their contributions to this overall investment vary from lower to higher grades? If yes, how?

Across grades, LLM-Development and TP-Validity were the most prominent predictors of learning time, while LLM-Integrative and TP-Importance were additional statistically significant predictors of this variable for Senior II students. From Senior I to Senior III students, the predictability of LLM-Development for learning time declined, whereas that of TP-Validity was obviously bigger for Senior III students than for the other two groups (see Figure 1).

When students’ average frequency of language learning practice served as the dependent variable, LLM-Integrative was the strongest predictor for Senior I (*β* = 0.23, *p* = 0.001) and Senior II (*β* = 0.29, *p* = 0.001), and was the second strongest predictor for Senior III (*β* = 0.28, *p* = 0.001). Hence across grades its predicting power was relatively stable. Differently, the contribution of TP-Validity notably increased across grades, ranging from being the third strongest predictor (*β* = 0.11, *p* = 0.001), to the second (*β* = 0.17, *p* = 0.001), and finally to the top (*β* = 0.29, *p* = 0.001). Interestingly, LLM-Development, which remained a statistically significant though somewhat secondary predictors of learning time for Senior II and III students, drastically diminished to be statistically non-significant for the last two groups for this criterion variable.

*RQ2*: For each grade, which LLM and TP variables constitute the strongest predictors of students’ engagement in each type of learning practice? Do their contributions to each type of learning practice vary from lower to higher grades? If yes, how?

In terms of specific types of learning practice, LLM-Integrative was found to be the most powerful predictor of LLP-Amusement across grades (*β* = 0.27–0.30, *p* = 0.001). It also kept its dominance in predicting LLP-Communicative (*β* = 0.27–0.30, *p* = 0.001). In addition, TP-Validity was another predictor of this type of learning which achieved statistical significance across three grades (*β* = 0.07–0.22, *p* = 0.032–0.001), with an apparent increase in predictability from lower to higher grades (see Figure 1). LLM-Requirement and TP-Importance, which were not statistically significant for the first two groups, also rose to achieve statistical significance for Senior III students, though their effect sizes were relatively small. It is worth noting that from Senior I to III, LLP-Development’s predictability of LLP-Communicative seemed to change in the negative direction (*β* = 0.03, −0.07, −0.18), indicating that as *Gaokao* approached, higher levels of development motivation actually inhibited students’ engagement in communicative learning practice.

Regarding LLP-Curriculum, it is clear that for Senior I students, LLM-Development was the top predictor (*β* = 0.32, *p* = 0.001). Nevertheless, its predictability decreased (*β* = 0.17, *p* = 0.001) and was surpassed by TP-Validity (*β* = 0.21, *p* = 0.001) for Senior II students. This trend further enlarged for Senior III students (see Figure 1). Beside these two major trends, we can see a rise of LLM-Integrative and a fall of TP-Importance in predicting this type of learning across the three grades (see Figure 1). Finally, four variables (i.e., LLM-Integrative, LLM-Development, TP-Validity and TP-Importance) more or less evenly accounted for students’ LLP-Test for Senior I students. As the grade level ascended, nevertheless, the predictability of TP-Validity significantly climbed (*β* = 0.19–0.26, *p* = 0.001), while that of LLM-Development quickly dropped to be statistically non-significant. The predicting power of TP-Importance was rather consistent and comparatively moderate in size across grades.

*RQ3*: For each grade, which LLM and TP variables constitute the strongest predictors of students’ reported achievement in English language learning? Do their contributions to reported achievement vary from lower to higher grades? If yes, how?

In terms of learning achievement, LLM-Integrative (*β* = 0.13, *p* = 0.001), LLM-Development (*β* = 0.15, *p* = 0.001) and TP-Validity (*β* = 0.10, *p* = 0.004) were the three biggest predictors of Senior I students’ reported English test scores. Aside from them, TP-Importance (*β* = 0.07, *p* = 0.034) was another statistically significant predictor for Senior II students. There was also a slight increase of LLM-Integrative’s effect size (*β* = 0.18, *p* = 0.001) and a significant drop of LLM-Development’s predicting power (*β* = 0.08, *p* < 0.037) compared with those for Senior I students. For Senior III students, only LLM-Integrative and TP-Validity (for both, *β* = 0.18, *p* = 0.001) were statistically significant predictors of this criterion variable. From lower to higher grades, we can see that, LLM-Integrative maintained its strength in predicting achievement, the predicting power of LLM-Development significantly decreased, while that of TP-Validity suddenly increased for the last student group.

## 4. Discussion

In this study, the effects of language learning motivations (LLMs) on Chinese high school students’ autonomous EFL learning after class were investigated and juxtaposed with those of test perceptions (TPs). As high schooling in China is notoriously influenced by the extremely high-stakes university entrance examination or *Gaokao* (Qi, 2005, 2007; Cheng and Qi, 2006), it was hypothesized that students’ out-of-class English learning would be strongly affected by their perceptions of the test, especially for Senior III students who are immediately faced with the test. Our findings generally confirmed these hypotheses, as there was an evident shift of motivational influence from more internalized LLMs (i.e., integrative and development motivations) to TP (i.e., perceived test validity) on both time investment and average learning frequency from lower to higher grades, suggesting an increased washback effect on students’ overall autonomous EFL learning as *Gaokao* approached (Zhan, 2009).

The results indicate that for Senior I students, who were probably the least affected by *Gaokao*, both integrative and development motivations were the major engines driving English learning outside class. This confirms the socio-educational model and related empirical findings regarding the beneficial effects of integrative orientation on autonomous learning (Wu, 2007; Csizér and Kormos, 2009; Gardner, 2010; Papi, 2010; Baleghizadeh and Rahimi, 2011). The significantly positive contribution from development motivation to overall learning effort also lends support to the assumption that internalized instrumental motives such as those for personal development or professional success share similarities with intrinsic motives, and would also motivate students to make sustained effort to learn (Dörnyei, 2005; Ryan and Deci, 2017).

Moving from overall learning to specific types of practice, the influence patterns of LLMs and TPs across grades differed from one type of practice to another. In general, it is clear that from lower to higher grades the effect of perceived test validity increased for three types of learning—communicative and curriculum-based learning as well as test drilling, suggesting that the more closely students were facing *Gaokao*, these three types of autonomous learning were increasingly affected by the test. While this is understandable for test drilling and even curriculum-based learning, as teaching content has been repeatedly found to be prone to the influence of highs-takes tests (Wall and Alderson, 1993; Qi, 2005, 2007), one may wonder why communicative learning, which involved non-test-related activities and was generally found to be free from instrumental and test-oriented motivations (Xie, 2015; Liu and Yu, 2021), was also significantly influenced by perceived test validity, particularly for higher-grade students who were closer to *Gaokao*. A possible explanation might be that the more students consider a language test to be a valid measure of language skills, the more they understand the importance of developing those skills in order to succeed in it, and would take actions accordingly.

It shall be noted that, despite the increased influence of perceived test validity, integrative motivation maintained its dominance in predicting communicative learning across the three student groups. This is different from curriculum-based learning, which showed a significantly decreased influence from development motivation and a considerably increased influence from perception of test validity. These suggest that communicative learning in general was less influenced by the approach of *Gaokao* than was curriculum-based learning. It may also be due to the fact that, as mentioned earlier, the content of teaching tends to be subject to the impact of high-stakes tests.

Another interesting finding is related to test drilling. Previous theories and empirical studies suggested that perceived test importance and instrumental goal orientations would be strong predictors of this type of learning (Ames, 1992; Green, 2007a; Senko et al., 2013; Liu and Yu, 2021), and this relation may be strengthened with the approach of test date (Zhan, 2009). However, our study revealed that the predictability of these two variables over test drilling was not as strong as expected, especially for the Senior III group. By contrast, perceived test validity was found to be the strongest determinant of Senior II and III students’ test drilling. This seems to partially support the finding from Xie and Andrews (2013), which showed that students’ endorsement of the construct of a test exerted greater influences than did their instrumental test motivations and perceived test importance on their test preparation practice.

Aside from its consistently significant prediction for communicative learning, integrative motivation was also the only dominant predicting variable for amusement learning across grades. This is consistent with the socio-educational theory, which posits that language learners with an integrative orientation are interested in the culture of the target language community (including songs, movies, and TV programs in the present case) and are willing to master the target language for the sake of communicating with its speakers (and thus the necessity to conduct communicative language learning activities; Gardner and Lambert, 1972; Gardner, 2010). Integrative motivation is normally long-term in nature, as mastering the target language and realizing social identification take time and sustainable efforts. This would explain why its predictive power for amusement and communicative learning appeared relatively stable and did not significantly diminish for students who were closer to the test.

In terms of students’ reported average scores in English language tests, integrative motivation was again among the top positive predictors across grades, while requirement motivation remained consistently nonsignificant. These are consistent with previous findings about the positive and little or even negative contributions of integrative or intrinsic and non-internalized instrumental motivations to learning outcome (Hernández, 2006; Baleghizadeh and Rahimi, 2011; McEown et al., 2014). One type of internalized instrumentality—development motivation—was also a significantly positive predictor of reported achievement, especially for Senior I students. However, its predictive power quickly declined for students from higher grades, as with its influences on overall learning effort (i.e., learning time and frequency). These seem to suggest that internalized instrumentality, though having the potential to prompt learners to spend effort on learning and make achievements in certain circumstances (Dörnyei, 2005; Ryan and Deci, 2017), may not be able to support learning as sustainably as integrative motivation would. This may also explain why this motivation was previously found to bear a positive but statistically nonsignificant relation with learning outcome (Shaikholeslami and Khayyer, 2006).

Contrary to the waning effect of development motivation, perceived test validity demonstrated a significantly increased positive effect on reported achievement from the first two grades to the Senior III group. It is likely that as *Gaokao* drew near, students who possess a positive conception of the test were more inclined to invest their time into preparing for it, including actively conducting English activities both directly and indirectly associated with the test (i.e., communicative, curriculum-based, and test practice). Some of these activities (such as communicative and curriculum-based practice) may have helped develop their language abilities. While activities such as test practice may have had little contribution to real language development, they may have helped students raise their test scores through enhanced test-taking skills (Farnsworth, 2013; Xie, 2013), resulting in higher reported achievements.

## 5. Limitations and implications

The results presented above should be interpreted with the study’s limitations in mind. The first major limitation is that the data was collected in 2014. Therefore, up-to-date data shall be collected in future to understand the status quo of Chinese high school EFL students’ learning motivations and test perceptions, as well their impact on learning behaviors and achievement.

Second, we did not longitudinally track the motivations, perceptions and learning of the same group of students. Instead, three student groups were simultaneously surveyed. Although a breakdown of the three groups’ backgrounds showed that they were rather similar, it cannot be guaranteed that there were other systematic differences which have confounded our results. Therefore, the patterns of the predictability of LLMs and TPs from lower to higher grades described in the present study are better regarded as being tentative. Future studies adopting a true longitudinal approach is warranted to further reveal the dynamics in the predictability of LLMs and TPs over language learning.

Third, due to the large number of students involved, we only asked them to report the range of their English test scores as a rough measure of their learning achievement. It is evident that this format largely limited the variance of this variable. Meanwhile, there was no guarantee of the validity, reliability and comparability of those school-based tests. Thereby, the relationships found between students’ reported score ranges and their LLMs and TPs may not represent the effects of those perception variables on real achievements in language learning. To further investigate such relationships, future studies may need to use valid test results or other reliable measures of language proficiency, or a combination of them.

Despite these limitations, we can draw a few important implications from the results of this study. The first is that for educational contexts in which high-stakes language tests play prominent roles, research into the motivational patterns behind language learning behaviors may benefit from a simultaneous investigation of the influences of both traditional language learning motivations and test-related motivations or perceptions. Although theories such as the socio-educational model and possible selves can also be said to have components which cover, either explicitly or implicitly, test-oriented motives (e.g., instrumental orientation and out-to L2 self), such components may not be able to explain the details about specific motives driving certain practice during certain periods of learning. As found in our study, students’ requirement motivation actually accounted for little variance in their reported overall and specific types of language learning across groups. This even applied to Senior III students, who were most immediately faced with *Gaokao* and thus were supposed to be most susceptible to instrumental goals. By contrast, perceived test validity explained the largest unique variance in Senior II and Senior III students’ test drilling and curriculum-based learning, indicating the utility of this specific test perception variable in accounting for students’ test-oriented practice.

Second, in modeling the relationship between motivational variables and learning practice, it is necessary to differentiate between different types of learning practice. Past studies concerning the relation between LLMs and language learning often focused on overall learning only, such as learning effort and overall frequency of strategy use (Gardner et al., 1997; Csizér and Kormos, 2009; Papi, 2010; Baleghizadeh and Rahimi, 2011; MacIntyre and Blackie, 2012), without further distinguishing the types of activities efforts were put into. It is evident in the present study that (a) the motivational profiles behind students’ overall learning were different from those behind specific types of learning practice and (b) the motives driving one type of activities differed from those driving another type. Therefore, future research on the effects of LLMs and even TPs on learning engagement may benefit from a more analytic rather than holistic view of language learning practice and probe into the motives behind task-specific effort expenditure.

## Data availability statement

The original contributions presented in the study are included in the article/supplementary material, further inquiries can be directed to the corresponding author.

## Ethics statement

The studies involving human participants were reviewed and approved by Shanghai International Studies University. Written informed consent to participate in this study was provided by the participants’ legal guardian/next of kin.

## Author contributions

XL was in charge of conceptualizing the study, data analysis, and drafting manuscript. MD was responsible for instrument development and validation, data collection, and manuscript revision. All authors contributed to the article and approved the submitted version.

## Funding

This study was funded by The Human Resources and Social Security Bureau of Shenzhen (Grant number: 2022TC0002).

## Conflict of interest

The authors declare that the research was conducted in the absence of any commercial or financial relationships that could be construed as a potential conflict of interest.

## Publisher’s note

All claims expressed in this article are solely those of the authors and do not necessarily represent those of their affiliated organizations, or those of the publisher, the editors and the reviewers. Any product that may be evaluated in this article, or claim that may be made by its manufacturer, is not guaranteed or endorsed by the publisher.
